# Impact of venetoclax‐based therapies on CLL‐associated autoimmune cytopenias: A retrospective study conducted by ERIC

**DOI:** 10.1002/hem3.70522

**Published:** 2026-11-06

**Authors:** Candida Vitale, Thomas Chatzikonstantinou, Christina Papangelou, Francesca Perutelli, Maria Chiara Montalbano, Giulia Bondielli, Elia Boccellato, Salem Alshemmari, Francesco Angotzi, Miguel Arguello‐Tomas, Tomas Arpas, Sandra Bašić‐Kinda, Sotiria Besikli‐Dimou, Łukasz Bołkun, Tomasz Buczek, Aleksandra Butrym, Katherine Chapis, Sofia Chatzileontiadou, Rosa Collado, Raul Cordoba Mascuñano, Lorenzo De Paoli, Marina Deodato, Maria Dimou, David Donaldson, Joanna Drozd‐Sokołowska, Salem Elias, Andrea Ferrario, Alberto Fresa, Anna Maria Frustaci, Rocío García‐Serra, Ajay Gogia, Isabel González‐Gascón‐y‐Marín, Vasko Graklanov, Maria Hryniewiecka, Zhu Huayuan, Elzbieta Iskierka‐Jażdżewska, Paula Jablonowska‐Babij, Ozren Jaksic, Elzbieta Kalicińska, Maria Kislova, Maya Koren‐Michowitz, Epameinondas Koumpis, Zuzana Kubová, Jorge Labrador, Pietro Lauzzana, Shai Levi, Francesca Martini, Mariia Mikhaleva, Eva Minga, Riccardo Moia, Elżbieta Morawiec‐Szymonik, Marta Morawska, Femke V. M. Mulder, Roberta Murru, Jacopo Olivieri, Ewa Paszkiewicz‐Kozik, Lata Rani, Josep Maria Roncero, Wojciech Salamon, Alessandro Sanna, Lydia Scarfò, Tereza Shokralla, Martin Šimkovič, Matevž Škerget, Emil Spasov, Antigoni Tranidou, Jagoda Tryc‐Szponder, Marzia Varettoni, Andrea Visentin, George Vrachiolias, David Waddell, Ewa Wasik‐Szczepanek, Kamil Wdowiak, Mateusz Wilk, Kamil Wiśniewski, Magdalena Witkowska, Mohamed Yassin, Seyma Yıldız, Klaudia Zielonka, Nil Albiol, Jennifer R. Brown, Benedetto Bruno, Mark Catherwood, Anastasia Chatzidimitriou, Michael Doubek, Gianluca Gaidano, Alessandro Gozzetti, Ritu Gupta, Eleftheria Hatzimichael, Yair Herishanu, Krzysztof Jamroziak, Ioannis Kotsianidis, Luca Laurenti, Eugene Nikitin, Maria Papaioannou, Tomáš Papajik, Martin Špaček, Niki Stavroyianni, Josephine M. I. Vos, Tomasz Wróbel, Münci Yağcı, Arnon P. Kater, Matthew Davids, Petra Langerbeins, Kostas Stamatopoulos, Paolo Ghia, Marta Coscia

**Affiliations:** ^1^ Division of Hematology, A.O.U. Città della Salute e della Scienza di Torino and Department of Molecular Biotechnology and Health Sciences University of Torino Torino Italy; ^2^ Institute of Applied Biosciences Centre for Research and Technology Hellas Thessaloniki Greece; ^3^ Department of Hematology Santa Croce e Carle Hospital Cuneo Italy; ^4^ Department of Medicine, Faculty of Medicine Kuwait University Kuwait City Kuwait; ^5^ Hematology Unit, Department of Medicine University of Padova Padova Italy; ^6^ Hematology Department, Hospital de la Santa Creu i Sant Pau, IIB‐Sant Pau and José Carreras Leukemia Research Institutes, Departamento de Medicina Universidad Autónoma de Barcelona Barcelona Spain; ^7^ Department of Internal Medicine ‐ Hematology and Oncology University Hospital Brno and Faculty of Medicine, Masaryk University Brno Czech Republic; ^8^ Division of Hematology, Department of Internal Medicine University Hospital Zagreb Zagreb Croatia; ^9^ Haematology St. George's University Hospital London United Kingdom; ^10^ Haematology Department Medical University Hospital Bialystok Poland; ^11^ Department of Hematology and Oncology Wroclaw Medical University Wroclaw Poland; ^12^ Oncology Support Centre for Clinical Trials Alfred Sokolowski Specialist Hospital in Walbrzych Walbrzych Poland; ^13^ Department of Medical Oncology Dana‐Farber Cancer Institute Boston Massachusetts United States; ^14^ Hematology Unit, 1st Department of Internal Medicine AUTH, AHEPA Hospital Thessaloniki Greece; ^15^ Servicio de Hematología Consorcio Hospital General Universitario de Valencia, Fundación de Investigación Hospital General Universitario de Valencia Valencia Spain; ^16^ Department of Hematology Fundacion Jimenez Diaz University Hospital, Health Research Institute IIS‐FJD Madrid Spain; ^17^ Division of Internal Medicine, Hematology Unit ASL Vercelli Vercelli Italy; ^18^ Department of Hematology, Niguarda Cancer Center ASST Grande Ospedale Metropolitano Niguarda Milano Italy; ^19^ Department of Hematology and Bone Marrow transplantation Unit National and Kapodistrian University of Athens Laikon General Hospital Athens Greece; ^20^ Clinical Haematology Belfast City Hospital Belfast United Kingdom; ^21^ Department of Hematology Transplantation and Internal Medicine Medical University of Warsaw Warsaw Poland; ^22^ Division of Hematology ASST Sette Laghi ‐ Ospedale di Circolo di Varese Varese Italy; ^23^ Hematology‐Oncology and Stem Cell Transplantation Unit, Department of Onco‐Hematology and Innovative Diagnostics Istituto Nazionale Tumori IRCCS Fondazione G. Pascale Napoli Italy; ^24^ Dr. B.R.A. IRCH, All India Institute of Medical Sciences (AIIMS) New Delhi India; ^25^ Hematology Department Infanta Leonor University Hospital Madrid Spain; ^26^ First Department of Internal Diseases Medical University of Plovdiv, Clinical Hematology Clinic, St. George University Hospital for Active Treatment Plovdiv Bulgaria; ^27^ Department of Hematology and Bone Marrow Transplantation Polish Red Cross Maritime Hospital Gdynia Poland; ^28^ Department of Hematology The First Affiliated Hospital of Nanjing Medical University Nanjing China; ^29^ Department of General Hematology and Internal Medicine Copernicus Memorial Hospital Lodz Poland; ^30^ Department of Hematology Medical University of Lodz Lodz Poland; ^31^ Department of Nursing, Faculty of Nursing and Midwifery Wroclaw Medical University Wroclaw Poland; ^32^ Department of Hematology Dubrava University Hospital Zagreb Croatia; ^33^ Clinical Department of Hematology, Cell Therapies and Internal Diseases Wroclaw Medical University Wroclaw Poland; ^34^ Department of Hematology, Oncology, and Chemotherapy, S. P. Botkin's City Hospital Russian Medical Academy of Continuous Professional Education Moscow Russia; ^35^ Department of Hematology, Shamir Medical Center, Zerifin and Sackler Faculty of Medicine Tel Aviv University Tel Aviv Israel; ^36^ Department of Haematology, Faculty of Medicine School of Health Sciences University of Ioannina Ioannina Greece; ^37^ Department of Hemato‐Oncology, Faculty of Medicine and Dentistry Palacký University and University Hospital Olomouc Olomouc Czech Republic; ^38^ Department of Hematology Hospital Universitario de Burgos, Instituto de Investigación Biosanitaria de Burgos (IBioBURGOS) Burgos Spain; ^39^ Hematology Clinic, ASUFC Udine Italy; ^40^ Department of Hematology, Tel Aviv Sourasky Medical Center and Faculty of Medicine Tel Aviv University Tel Aviv Israel; ^41^ Strategic Research Program on CLL Comprehensive Cancer Center, IRCCS Ospedale San Raffaele Milano Italy; ^42^ Division of Hematology, Department of Translational Medicine Università del Piemonte Orientale and A.O.U. Maggiore della Carità Novara Italy; ^43^ Department of Internal Medicine and Oncological Chemotherapy Andrzej Mielęcki Independent Public Clinical Hospital Katowice Poland; ^44^ Experimental Hematooncology Department Medical University of Lublin Lublin Poland; ^45^ Sanquin Research and Landsteiner Laboratory of the Academic Medical Center Amsterdam UMC, University of Amsterdam Amsterdam The Netherlands; ^46^ Hematology and Stem Cell Transplantation Unit Ospedale Oncologico A. Businco, ARNAS “G. Brotzu” Cagliari Italy; ^47^ Department of Lymphoid Malignancies Maria Sklodowska‐Curie National Research Institute of Oncology Warsaw Poland; ^48^ Department of Hematology Institut Català d'Oncologia (ICO)‐Hospital Universitari Dr. Josep Trueta, Girona and Haematology Research Group, Institut d'Investigació Biomèdica de Girona Dr. Josep Trueta Girona Spain; ^49^ Hematology Unit, Department of Oncology AOU Careggi Firenze Italy; ^50^ School of Medicine Università Vita‐Salute San Raffaele Milano Italy; ^51^ First Faculty of Medicine, 1st Department of Medicine ‐ Hematology Charles University and General Hospital in Prague Prague Czech Republic; ^52^ 4th Department of Internal Medicine – Haematology, Faculty of Medicine in Hradec Králové University Hospital and Charles University in Prague Hradec Kralove Czech Republic; ^53^ Department of Haematology and University of Ljubljana, Faculty of Medicine University Medical Centre Ljubljana Ljubljana Slovenia; ^54^ University Hospital “Sveti Georgi”, Medical University ‐ Plovdiv Plovdiv Bulgaria; ^55^ Department of Hematology and Bone Marrow Transplantation Poznan University of Medical Sciences Poznan Poland; ^56^ Division of Hematology Fondazione IRCCS Policlinico San Matteo Pavia Italy; ^57^ Department of Hematology University Hospital of Alexandroupolis Democritus University of Thrace Alexandroupolis Greece; ^58^ Department of Hematooncology and Bone Marrow Transplantation Medical University in Lublin Lublin Poland; ^59^ Department of Internal Medicine and Oncological Chemotherapy, Faculty of Medical Sciences in Katowice Medical University of Silesia Katowice Poland; ^60^ Haematology Clinical Department University Hospital in Cracow Cracow Poland; ^61^ Department of Hematology Institute of Hematology and Transfusion Medicine Warsaw Poland; ^62^ Hematology Section, Department of Medical Oncology National Center for Cancer Care and Research Doha Qatar; ^63^ Department of Hematology Gazi University Medical Faculty Ankara Turkey; ^64^ Central European Institute of Technology (CEITEC) Masaryk University Brno Czech Republic; ^65^ Division of Hematology University of Siena Siena Italy; ^66^ Department of Diagnostic Imaging, Radiation Oncology and Hematology Fondazione Policlinico Universitario Agostino Gemelli IRCCS Roma Italy; ^67^ Section of Hematology, Department of Radiological and Hematological Sciences Catholic University of Sacred Heart Roma Italy; ^68^ Hematology Department and HCT Unit G Papanicolaou Hospital Thessaloniki Greece; ^69^ Department of Hematology Cancer Center Amsterdam, Amsterdam University Medical Center, University of Amsterdam Amsterdam The Netherlands; ^70^ Department I of Internal Medicine and German CLL Study Group, Center for Integrated Oncology Aachen Bonn Cologne Duesseldorf University of Cologne, Faculty of Medicine and University Hospital Cologne Cologne Germany; ^71^ Division of Hematology, ASST Sette Laghi ‐ Ospedale di Circolo di Varese and Department of Medicine and Surgery University of Insubria Varese Italy

## Abstract

The impact of venetoclax‐based therapies on autoimmune cytopenias (AIC) in patients with chronic lymphocytic leukemia (CLL) is largely unknown. This study aimed to evaluate preexisting and treatment‐emergent AIC in patients with CLL treated with venetoclax, focusing on the differential impact of venetoclax monotherapy versus venetoclax‐based combinations. In this retrospective multicenter study, data from 1720 patients were collected; 23.1% received venetoclax single agent and 76.9% venetoclax combinations (mostly containing anti‐CD20 monoclonal antibodies and/or BTK inhibitors). Combination regimens were used in 97.7% of treatment‐naïve (TN) patients and 65.8% of patients with relapsed/refractory (R/R) CLL. Preexisting AIC were reported in 11.7% of patients (8.7% in TN and 13.4% in R/R, P = 0.005). CLL‐related genetic risk factors and disease outcomes did not differ in patients with and without preexisting AIC. Irrespective of treatment type, venetoclax therapy resulted in AIC resolution or improvement in 63% of patients with active or controlled AIC. Treatment‐emergent AIC occurred in 2.6% of patients (2% in TN and 2.9% in R/R, not statistically significant). AIC episodes were reported in 4.3% of patients treated with venetoclax monotherapy and in 2.1% of those treated with venetoclax‐based combinations (P = 0.02). After standard management, AIC was considered resolved or controlled in 82% of the patients, with no substantial difference between treatment groups. Venetoclax‐based therapies have a beneficial impact on the outcome of CLL‐associated preexisting AIC. Treatment‐emergent AIC is a rare and generally manageable event. Although more frequent with venetoclax monotherapy in unadjusted analysis, this association was attenuated after adjustment for baseline prognostic factors.

## Graphical abstract

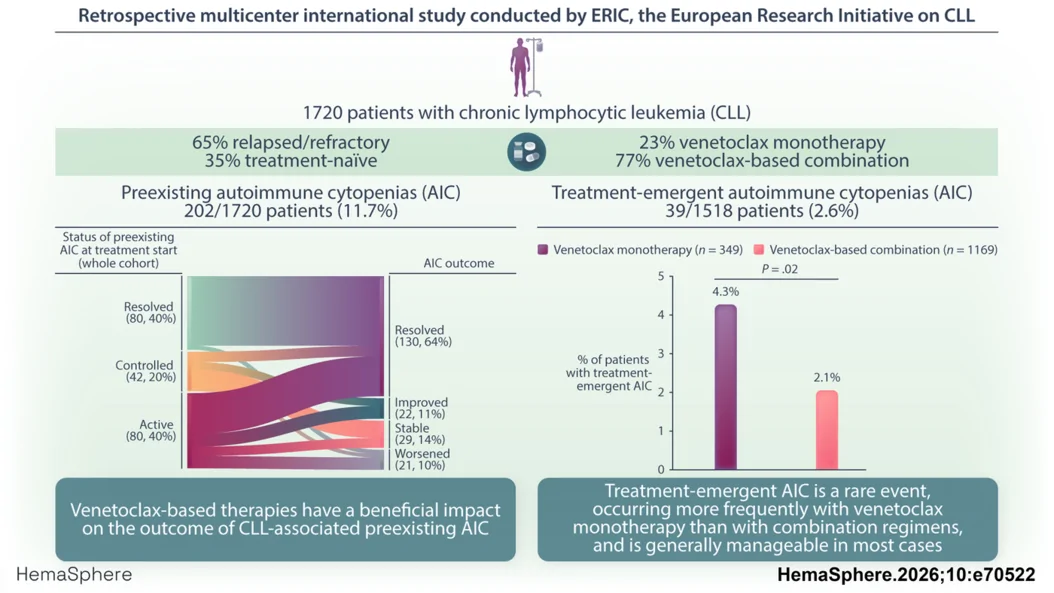

## INTRODUCTION

Chronic lymphocytic leukemia (CLL) is associated with various immune system disturbances that progressively worsen during the disease course.^1^ This immune imbalance increases the risk of infections, secondary cancers, and autoimmune disorders, complicating both the disease course and treatment.^2^, ^3^, ^4^ Autoimmune cytopenias (AIC) occur in 5%–13% of CLL cases, manifesting as autoimmune hemolytic anemia (AIHA), immune thrombocytopenia (ITP), or, very rarely, pure red blood cell aplasia (PRCA) or autoimmune granulocytopenia (AIG).^5^, ^6^, ^7^, ^8^, ^9^ Treatment for AIC in CLL usually targets the autoimmune condition itself (i.e., steroid‐based in warm AIHA and ITP; rituximab‐based in cold AIHA). These approaches usually result in high response rates.^10^ However, recurrences are common, and patients who are refractory to therapy or show additional signs of CLL progression are generally treated with CLL‐directed therapies.^11^

In recent years, targeted therapies, including venetoclax, have been introduced into the therapeutic landscape of CLL, demonstrating remarkable efficacy.^12^ However, their impact on CLL‐related AIC remains poorly understood, primarily because patients with active AIC are generally excluded from clinical trials, and few studies have specifically explored the role of targeted drugs in this context.^9^, ^13^, ^14^, ^15^, ^16^, ^17^ Additionally, there are currently no established guidelines for managing AIC that arise during treatment with targeted agents.

We previously investigated the impact of targeted therapies on the natural history of CLL‐associated AIC. In our analysis, ibrutinib, idelalisib, and venetoclax were all associated with a beneficial effect on preexisting AIC.^9^ Notably, treatment‐emergent AIC occurred more frequently in patients receiving venetoclax—primarily administered as monotherapy—compared to those treated with ibrutinib or idelalisib.^9^ The observation that treatment‐emergent AIC did not develop in a small subset of patients who were receiving venetoclax in combination with rituximab suggested that the addition of an anti‐CD20 monoclonal antibody (mAb) may exert a protective effect against the development of AIC. Consistently, in the Phase III Murano trial, only three patients discontinued treatment due to AIC in the venetoclax plus rituximab arm,^18^ and in the CLL14 trial, no AIC episodes were reported in patients receiving venetoclax plus obinutuzumab.^19^ More recently, two patients with CLL‐related refractory AIHA were reported to be successfully managed with venetoclax plus rituximab.^20^ Similarly, double or triple combinations of venetoclax plus BTK inhibitors (BTKi) (i.e., ibrutinib, acalabrutinib, and zanubrutinib), administered with or without an anti‐CD20 mAb, appear to be safe, with only one case of AIHA reported in a Phase II trial investigating the efficacy of ibrutinib plus venetoclax.^21^, ^22^, ^23^, ^24^, ^25^

Based on this rationale, the objective of this study was to evaluate in detail the clinical characteristics, incidence, evolution, and outcomes of two distinct clinical settings: (i) preexisting AIC in patients with CLL undergoing treatment with venetoclax‐containing regimens, and (ii) newly occurring treatment‐emergent AIC developing during venetoclax‐based therapies. We specifically aimed at addressing the differential impact of continuous treatment with venetoclax monotherapy compared to fixed‐duration venetoclax‐based combination regimens.

## METHODS

This is a retrospective multicenter international study conducted by ERIC, the European Research Initiative on CLL. Investigators at each participating site provided data on consecutive patients treated with venetoclax‐based regimens for CLL/small lymphocytic lymphoma (SLL). The study was conducted in accordance with the Declaration of Helsinki and was approved by the local ethics committees. Data were collected through chart review by the local teams and included—when available—baseline demographics, date of CLL diagnosis and disease history including previous treatments, immunoglobulin heavy variable (IGHV) gene somatic hypermutation status, cytogenetic status for chromosomes 11q, 13q, 17p, and 12 determined by fluorescence in situ hybridization, *TP53* and *NOTCH1* gene mutation status, comorbidities and cumulative illness rating scale (CIRS) score, β_2_‐microglobulin level, presence of hypogammaglobulinemia, results of direct antiglobulin test (DAT) and anti‐platelet antibodies test.

AIC were diagnosed and managed according to the treating physician's judgment, but reference definitions of AIC, based on commonly accepted criteria for CLL‐associated AIC^10^ and consistent with those used in our previous study,^9^ were shared among investigators to categorize preexisting and treatment‐emergent cases, as well as their status and outcome (Tables S1 and S2). In the whole cohort, preexisting AIC were defined as episodes occurring before the start of the venetoclax‐based regimen, regardless of their status at treatment start. In patients with no preexisting AIC, treatment‐emergent AIC were defined as episodes occurring at any time during treatment with venetoclax‐based therapy.

The response of CLL to treatment was defined according to the International Workshop on Chronic Lymphocytic Leukemia (iwCLL) guidelines.^11^ Bone marrow biopsy was not uniformly performed across participating centers to confirm CR.

Descriptive statistics were used to summarize patients' characteristics. Follow‐up was defined as the time from start of venetoclax‐based treatment to last follow‐up date. Progression‐free survival (PFS) was defined as the time from start of venetoclax‐based treatment to disease progression or death from any cause, the earliest of these two events. Overall survival (OS) was defined as the time from start of venetoclax treatment to death. Cases that did not experience the event of interest (e.g., progression or death) were censored at the last follow‐up date. PFS and OS were depicted using the Kaplan–Meier method, and univariate and multivariate associations with survival outcomes were evaluated using Cox proportional hazards regression analysis. Time until treatment‐emergent AIC was defined as the time from venetoclax start until treatment‐emergent AIC onset.

The patient‐time incidence rate of treatment‐emergent AIC in each treatment group was estimated by dividing the number of AIC episodes by the total number of years the patients were at risk (i.e., venetoclax exposure).

Associations between variables were evaluated using the Pearson correlation coefficient.

Statistical analyses were performed using R version 4.4.2. The significance level was set to 5%.

## RESULTS

### Characteristics of patients

Data from 1720 patients with CLL treated with venetoclax‐containing regimens were collected from 56 centers in 19 different countries. The characteristics of the patients at the start of venetoclax treatment are summarized in Table 1. The median age was 68 years (range, 27–92), the median CIRS score was 6 (range, 0–30), and 551 patients (44.7%) were staged Binet C.

**Table 1:** Characteristics of patients at the time of venetoclax‐based treatment initiation.

	Whole cohort		Treatment‐emergent AIC		No treatment‐emergent AIC	
	No. evaluable patients		No. evaluable patients		No. evaluable patients	
Male sex, number (%)	1720	1156 (67.2%)	39	29 (74.4%)	1479	979 (66.2%)
Age, years, median (range)	1720	68 (27–92)	39	69 (39–86)	1461	68 (27–92)
Binet stage, number (%)	1233		34		1035	
A		172 (13.9%)		4 (11.8%)		145 (14%)
B		510 (41.4%)		8 (23.5%)		465 (44.9%)
C		551 (44.7%)		**22** (**64.7%)**		**425** (**41.1%)** §
CIRS score, median (range)	1035	6 (0–30)	31	6 (0–13)	875	5 (0–30)
β2‐Microglobulin, mg/L, median (range)	728	3.9 (0.9–17.9)	14	4.1 (1.1–10)	612	3.9 (0.9–17.9)
Unmutated IGHV status, number (%)	1005	718 (71.4%)	22	17 (77.3%)	857	614 (71.6%)
Del(13q), number (%)	997	493 (49.4%)	24	12 (50%)	849	407 (47.9%)
Del(11q), number (%)	1134	302 (26.6%)	25	9 (36%)	963	253 (26.3%)
Tris(12), number (%)	960	196 (20.4%)	23	4 (17.4%)	814	174 (21.4%)
Del(17p), number (%)	1256	236 (18.8%)	27	9 (29.6%)	1066	197 (18.5%)
Mutated TP53, number (%)	917	178 (19.4%)	23	8 (34.8%)	782	148 (18.9%)
*TP53* aberration, number (%)	921	314 (34.1%)	25	**13** (**52%)**	778	**258** (**33.2%)** °
Hypogammaglobulinemia (IgG < 300 mg/dL), number (%)	875	94 (10.7%)	18	**5** (**27.8%)**	729	**70** (**9.6%)** *
DAT positivity	722	130 (18%)	23	**9** (**39.1%)**	595	**55** (**9.2%)** #
DAT specificity, number (%)	98		5		32	
Anti‐IgG		59 (60.2%)		2 (40%)		22 (68.8%)
Anti‐C3		6 (6.1%)		0		4 (12.5%)
Anti‐IgG and anti‐C3		31 (31.6%)		3 (60%)		6 (18.8%)
Other		2 (2%)		0		0
History of other type of autoimmune phenomena, number (%)	1019	74 (7.3%)	22	2 (9.1%)	997	72 (7.2%)
Treatment‐naïve patients, number (%)	1720	598 (34.8%)	39	11 (28.2%)	1479	536 (36.2%)
Number of previous therapies, median (range)	1720	1 (0–13)	39	2 (0–9)	1479	1 (0–13)

*Note*: Categorical variables were compared using the *χ* ^2^ test and continuous variables using Student's *t*‐test. Only statistically significant P‐values (in bold) are reported; all other comparisons were not statistically significant (NS).

Abbreviations: AIC, autoimmune cytopenia; CIRS, cumulative illness rating scale; DAT, direct antiglobulin test; IGHV, immunoglobulin heavy variable.

^§^ P = 0.006.

^°^ P = 0.049.

* P = 0.01.

^#^ P < 0.001.

Overall, 65.2% of patients had been previously treated, with a median number of previous treatment lines of 1. Only 67 out of 1121 patients (6%) with relapsed/refractory (R/R) disease had been previously treated with targeted agents only. In terms of biological prognostic factors, 71.4% of patients had unmutated IGHV status, 18.8% carried del(17p), and 19.4% a *TP53* mutation. Among patients tested, the DAT was positive in 18%, and 7.3% of the whole cohort had a documented history of other autoimmune conditions (excluding AIC). The most frequently reported disorders were autoimmune thyroid diseases (33.8% of cases, including Hashimoto's thyroiditis, Graves–Basedow disease and unspecified autoimmune thyroid disease), followed by psoriasis (12.2%) and rheumatoid arthritis (8.1%).

### Treatment received and outcome

Venetoclax was prescribed as a single agent in 398 patients (23.1%) and in combination in 1322 (76.9%). Specific treatment combinations received by patients are described in Table 2. Specifically, in the treatment‐naïve (TN) cohort, 14/598 patients (2.3%) received venetoclax single agent, and among patients who received a venetoclax‐based combination, the vast majority (88.9%) received venetoclax plus an anti‐CD20 mAb (62.8% obinutuzumab and 26% rituximab) and 9.7% received a venetoclax‐based combination containing a BTKi with or without an anti‐CD20 mAb (6.1% ibrutinib and 3.6% acalabrutinib). In the R/R cohort, 384/1122 patients (34.2%) received venetoclax single agent, and among those who were treated with a venetoclax‐based combination, 90.8% received venetoclax plus an anti‐CD20 mAb (87.4% rituximab and 3.4% obinutuzumab) and 6% received a venetoclax‐based combination containing a BTKi with or without an anti‐CD20 mAb (4% ibrutinib, 0.5% acalabrutinib, 1.5% pirtobrutinib).

**Table 2:** Description of treatment received by patients.

All patients, *n* = 1720	Venetoclax single agent: 398 (23.1%)	Rituximab: 797 (60.3%)
	Venetoclax‐based combination: 1322 (76.9%)	Obinutuzumab: 392 (29.7%)
		Ibrutinib: 42 (3.2%)
		Ibrutinib + rituximab: 1 (0.1%)
		Ibrutinib + obinutuzumab: 23 (1.7%)
		Acalabrutinib: 13 (1%)
		Acalabrutinib + obinutuzumab: 12 (0.9%)
		Pirtobrutinib + rituximab: 11 (0.8%)
		Other: 31 (2.3%)
Treatment‐naïve patients, *n* = 598	Venetoclax single agent: 14 (2.3%)	Rituximab: 152 (26%)
	Venetoclax‐based combination: 584 (97.7%)	Obinutuzumab: 367 (62.9%)
		Ibrutinib: 13 (2.2%)
		Ibrutinib + obinutuzumab: 23 (3.9%)
		Acalabrutinib: 12 (2.1%)
		Acalabrutinib + obinutuzumab: 9 (1.5%)
		Other: 8 (1.4%)
Relapsed/refractory patients, *n* = 1122	Venetoclax single agent: 384 (34.2%)	Rituximab: 645 (87.4%)
	Venetoclax‐based combination: 738 (65.8%)	Obinutuzumab: 25 (3.4%)
		Ibrutinib: 29 (3.9%)
		Ibrutinib + rituximab: 1 (0.1%)
		Acalabrutinib: 1 (0.1%)
		Acalabrutinib + obinutuzumab: 3 (0.4%)
		Pirtobrutinib + rituximab: 11 (1.5%)
		Other: 23 (3.1%)

For the entire cohort, the median follow‐up from the start of venetoclax‐based treatment until last observation or death was 21.8 (95% CI: 19.5–21.9) months (TN patients: 13.4 months, 95% CI 11.4–16.6; R/R patients: 20.4 months, 95% CI 19.9–23.6). The median duration of treatment was 12 months (interquartile range [IQR] 6.4–23.8) (TN patients: 11 months, IQR 6.39–13.13; R/R patients: 14.9 months, IQR 7.52–24.7). Overall, 991 patients of 1613 for whom data were available (61.4%) discontinued treatment (58.1% of TN patients and 62.7% of R/R patients). Reasons for treatment discontinuation included treatment plan in 47.8% (76.6% for TN patients and 32.3% for R/R patients), toxicity in 16.7% (12.2% for TN patients and 19.3% for R/R patients), treatment failure in 16.1% (2.1% for TN patients and 22.7% for R/R patients), and death in 7.8% (2.7% for TN patients and 10.2% for R/R patients) (data available for 987 patients).

The overall response rate (ORR) was 91.7%, with a 45.1% rate of complete response or complete response with incomplete marrow recovery (CR/CRi) (data available for 1566 patients). ORR and CR/CRi rates were 94.9% and 50.8% for TN patients, and 88.9% and 41.6% for R/R patients, respectively. Median PFS for TN and R/R patients was 71.5 (95% CI: 65.4–89) and 48.4 (95% CI: 44.5–56.5) months, respectively, and median OS was not reached and 65.3 (95% CI: 59.9–85.6) months, respectively (Figure 1). In the TN cohort, for the 97.7% of patients who received a venetoclax‐based fixed‐duration combination, median PFS was 82.1 (95% CI: 65.8–not estimable) months, whereas the median OS was not reached. In the R/R cohort, patients receiving venetoclax monotherapy had a median PFS of 41.1 (95% CI: 34–47.7) months and a median OS of 51.2 (95% CI: 43.1–65.2) months, whereas patients receiving a time‐limited venetoclax‐containing combinations overall showed a median PFS of 53.6 (95% CI: 4.62–65.3) months and a median OS of 81.4 (95% CI: 62.3–not estimable) months.

**Figure 1:**
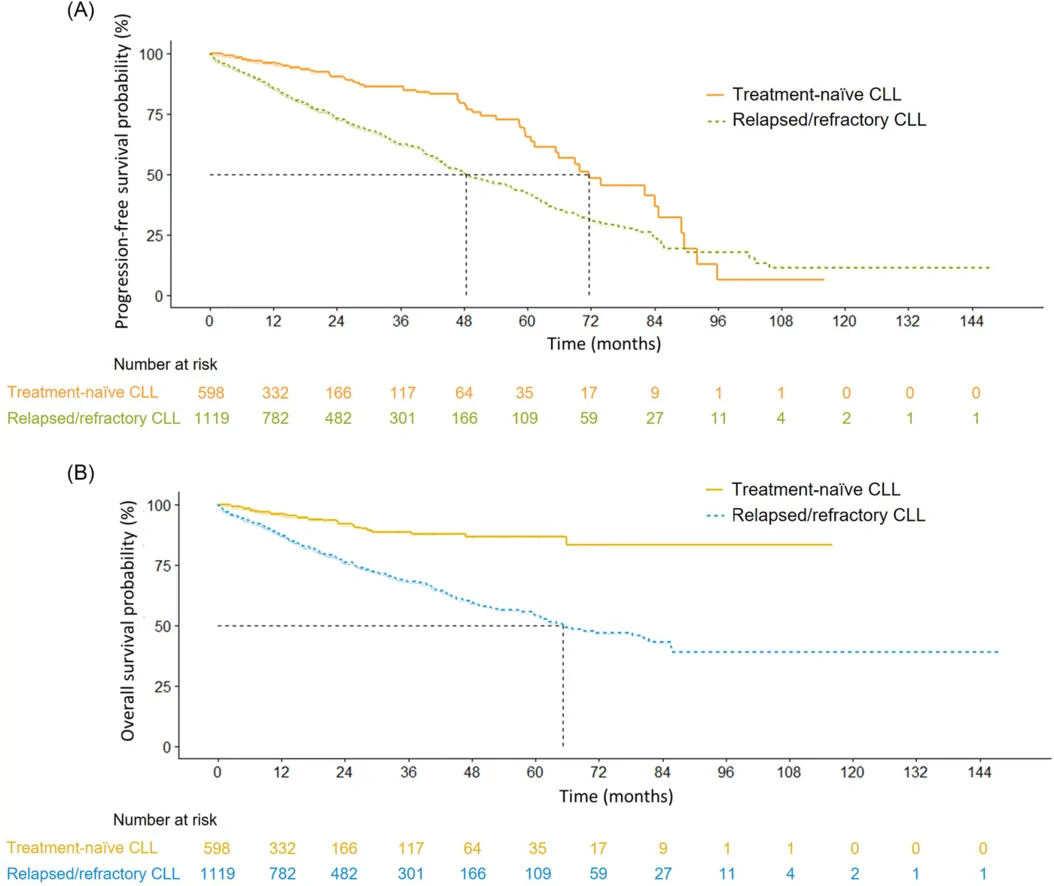
Progression‐free survival (PFS) (A) and overall survival (OS) (B) in patients with treatment‐naïve and relapsed/refractory chronic lymphocytic leukemia (CLL) treated with venetoclax‐containing regimens.

### Preexisting AIC

A history of preexisting AIC was reported in 202 patients (11.7%), of whom 55% had AIHA, 31.2% ITP, 9.9% AIHA and ITP (Evans syndrome), 1.5% PRCA, 0.5% AIHA and PRCA, 0.5% AΙG and 1.5% ITP and AΙG. Patients receiving venetoclax‐based therapy in the R/R setting had a significantly higher rate of preexisting AIC (150/1122, 13.4%) compared to those treated in the frontline setting (52/598, 8.7%) (P = 0.005).

Before starting treatment with venetoclax, 170/202 (84.1%) patients with preexisting AIC had received an AIC‐directed treatment, mostly consisting of steroids only (42.9%), steroids + rituximab (13.5%), or multiple treatments (29.4%). The characteristics of patients with and without preexisting AIC at the start of venetoclax‐based treatment are depicted in Table S3. The percentage of patients receiving venetoclax as a frontline treatment was significantly higher in patients without preexisting AIC compared to those with a preexisting AIC (36% vs. 25.7%, P = 0.005). As expected, the rate of baseline DAT positivity was significantly higher among patients with a history of preexisting AIC compared to those without (63.5% vs. 10.4%, P < 0.001). In the overall cohort, patients with and without preexisting AIC did not differ significantly with respect to CLL‐associated genetic prognostic factors (Table S3), and similar findings were observed when TN and R/R patients were analyzed separately (Tables S4 and S5). Similarly, no significant differences in ORR, CR/CRi rate (data not shown), PFS, or OS were observed either in the overall cohort or after stratification according to treatment setting (Figure 2). Additional multivariable Cox regression analyses performed separately in TN and R/R patients are reported in Tables S6 and S7. Median treatment duration was comparable between patients with and without preexisting AIC (13.3 and 12.3 months, respectively), as were venetoclax discontinuation rates (62.7% and 61.3%, respectively). However, patients with preexisting AIC had a higher rate of treatment discontinuation due to toxicity compared to patients with no preexisting AIC (25% of patients who discontinued vs. 15.6%, respectively; P = 0.009).

**Figure 2:**
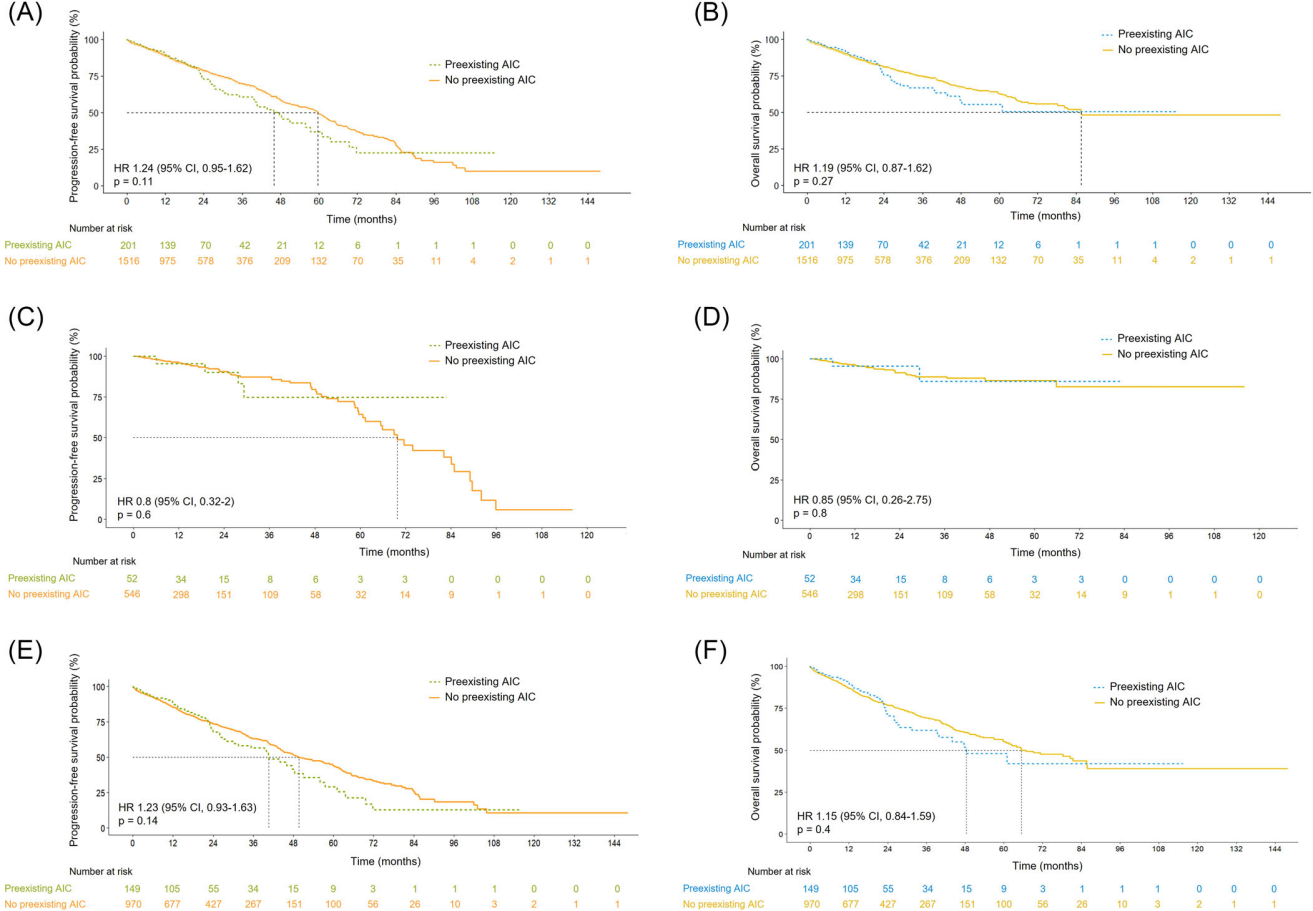
**Progression‐free survival (PFS) and overall survival (OS) in patients with and without preexisting autoimmune cytopenias (AIC).** Survival analyses are shown for the overall cohort **(A, B)** and separately for treatment‐naïve **(C, D)** and relapsed/refractory **(E, F)** patients. In the overall cohort, median PFS was 45.9 months and 59.7 months for patients with and without preexisting AIC, respectively **(A)**. Median OS was not reached and was 85.6 months for patients with and without preexisting AIC, respectively **(B)**. Hazard ratios (HRs) and 95% confidence intervals (CIs) derived from univariate Cox regression analyses are reported in each panel.

The status of preexisting AIC at the start of treatment with a venetoclax‐based therapy and evolution during treatment is depicted in Figure 3. In the whole cohort, at treatment initiation, AIC was active in 40%, controlled in 20%, and resolved in 40% of patients. Venetoclax‐based treatment induced AIC resolution or improvement in 73% of patients with active AIC, and 45% of patients with controlled AIC. Considering patients treated with venetoclax monotherapy and with venetoclax‐based combinations as two separate groups, at treatment initiation, AIC was active in 25% and 44%, controlled in 18% and 22%, and resolved in 57% and 34% of patients, respectively. Venetoclax single agent induced AIC resolution or improvement in 83% of patients with active AIC, and in 33% of patients with controlled AIC. Venetoclax‐based combinations induced AIC resolution or improvement in 71% of patients with active AIC, and 48% of patients with controlled AIC.

**Figure 3:**
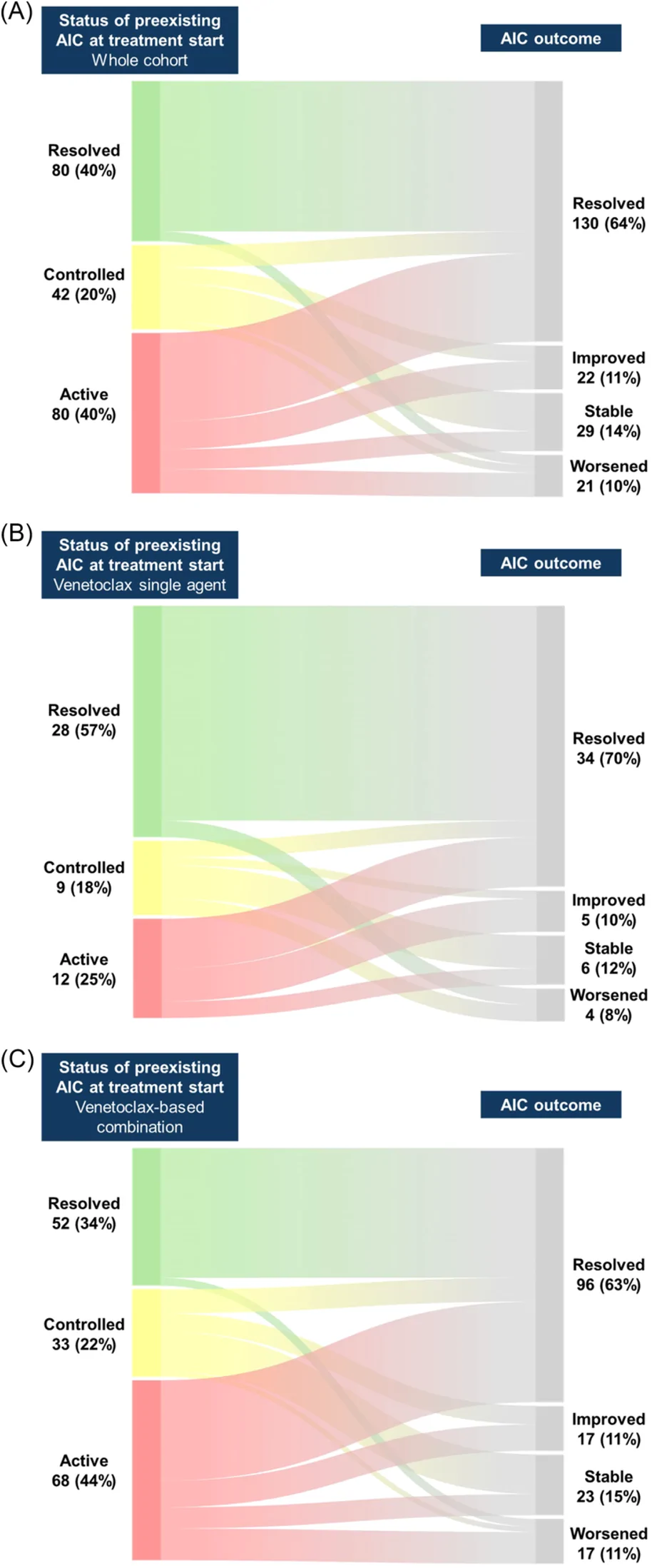
**Evolution of preexisting autoimmune cytopenias (AIC) during treatment with venetoclax‐based regimens.** The status of preexisting AIC at the start of treatment and their evolution during therapy is shown for the whole cohort **(A)**, for venetoclax monotherapy group **(B)**, and for patients receiving a venetoclax‐based combination **(C)**. For each patient group, stratified according to AIC status at treatment initiation (i.e., resolved, controlled, or active), the number and percentage of patients with different outcomes (i.e., resolved, improved, stable, or worsened) are reported.

### Frequency and characteristics of treatment‐emergent AIC

Amongst 1518 patients with no prior history of AIC receiving venetoclax‐based therapy, there were 39 cases (2.6%) of treatment‐emergent AIC, with a median onset time of 5.9 (IQR 2.11–7.3) months from treatment initiation. The frequency of treatment‐emergent AIC was not significantly different in TN patients compared to R/R (2% vs. 2.9%, respectively).

Among patients with a new‐onset AIC, AIHA was reported in 15 cases (38.5%), ITP in 20 (51.3%), Evans syndrome in 3 (7.7%) and PRCA in 1 (2.5%). The baseline characteristics of patients with and without treatment‐emergent AIC during venetoclax‐based treatment are presented in Table 1. Of note, in the subgroup of patients who developed treatment‐emergent AIC, we observed a significantly higher proportion of cases with *TP53* aberration (del(17p) and/or *TP53* mutation) compared with patients who did not develop AIC (52% vs. 33.2%, P = 0.049). Similarly, hypogammaglobulinemia was more frequent among patients with treatment‐emergent AIC (27.8% vs. 9.6%, P = 0.01), as was DAT positivity (39.1% vs. 9.2%, P < 0.001). Additionally, a significantly higher proportion of patients with treatment‐emergent AIC had a Binet stage C disease at the time of treatment initiation (64.7% vs. 41.1%, P = 0.006).

The frequency of new‐onset, treatment‐emergent AIC was lower among patients receiving venetoclax‐based combinations compared with those treated with venetoclax monotherapy (2.1% vs. 4.3% of patients, P = 0.02) (Figure 4). Analysis of individual combination subgroups showed a similarly reduced risk of developing newly occurring AIC compared with venetoclax monotherapy in patients treated with venetoclax plus an anti‐CD20 mAb (rituximab or obinutuzumab) (2.2% of patients), with an even more pronounced reduction in those receiving venetoclax combined with a BTKi, with or without anti‐CD20 mAb (1/101 patients, 1%). To account for potential baseline imbalances between treatment groups, additional multivariable logistic regression analyses were performed (Table S8). After adjustment for the main available prognostic variables, the association between venetoclax monotherapy and the occurrence of treatment‐emergent AIC was attenuated and no longer reached statistical significance (OR 2.23, 95% CI 0.89–5.57; P = 0.086).

**Figure 4:**
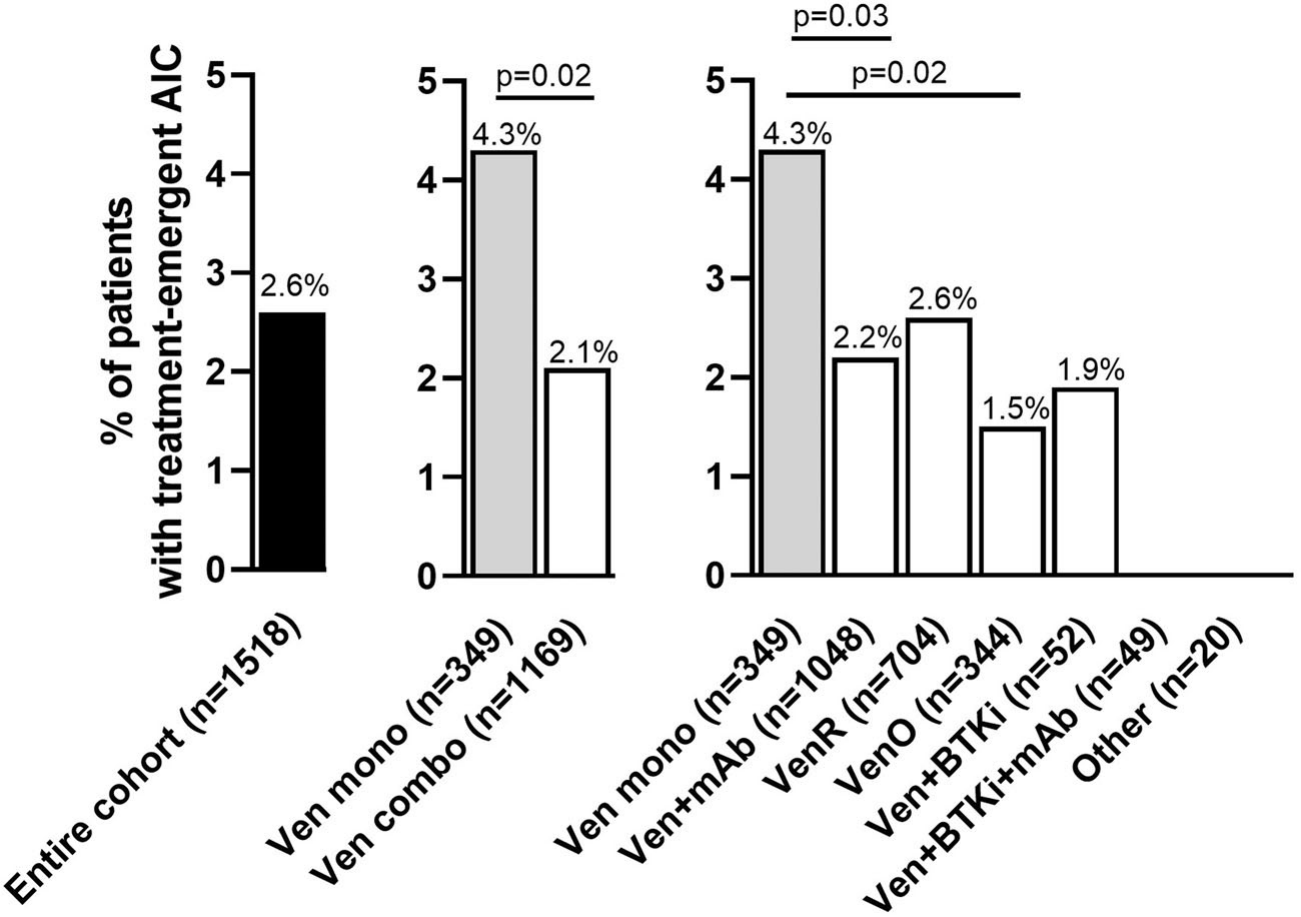
**Frequency of treatment‐emergent autoimmune cytopenias (AIC) in different treatment groups.** BTKi, BTK inhibitors; mAb, anti‐CD20 monoclonal antibody; Ven combo, venetoclax‐based combination; Ven mono, venetoclax monotherapy; VenO, venetoclax + obinutuzumab; VenR, venetoclax + rituximab.

Also, when we looked at the estimated incidence rate of treatment‐emergent AIC, we found no difference between patients receiving fixed‐duration combinations containing venetoclax compared to patients receiving continuous venetoclax single agent. Indeed, the incidence rate of treatment‐emergent AIC was 20 episodes per 1000 patients per year of exposure to venetoclax monotherapy and 16 episodes per 1000 patients per year of exposure to a venetoclax combination.

### Outcome of patients with treatment‐emergent AIC

We next compared patients with and without treatment‐emergent AIC in terms of outcomes and tolerability of venetoclax‐based therapy. The ORR of venetoclax‐based therapy was similar between the two groups (91.4% for patients with treatment‐emergent AIC and 92% for those without), whereas the CR/CRi rate was significantly lower in patients with treatment‐emergent AIC (22.9% vs. 45.5%, P = 0.006). Notably, treatment duration and discontinuation rates were comparable between the two groups. Finally, at the present follow‐up, patients who did or did not develop a treatment‐emergent AIC had no statistically significant differences in PFS (median PFS: 44.8 [95% CI: 29.1–not estimable] and 59.9 [95% CI: 55.9–65.3] months, respectively) and OS (median OS: 49.7 [95% CI: 33.7–not estimable] and 85.6 [95% CI: 80.5–not estimable] months, respectively) (Figure 5). Similarly, multivariable Cox regression analysis confirmed that treatment‐emergent AIC was not independently associated with OS (Table S9).

**Figure 5:**
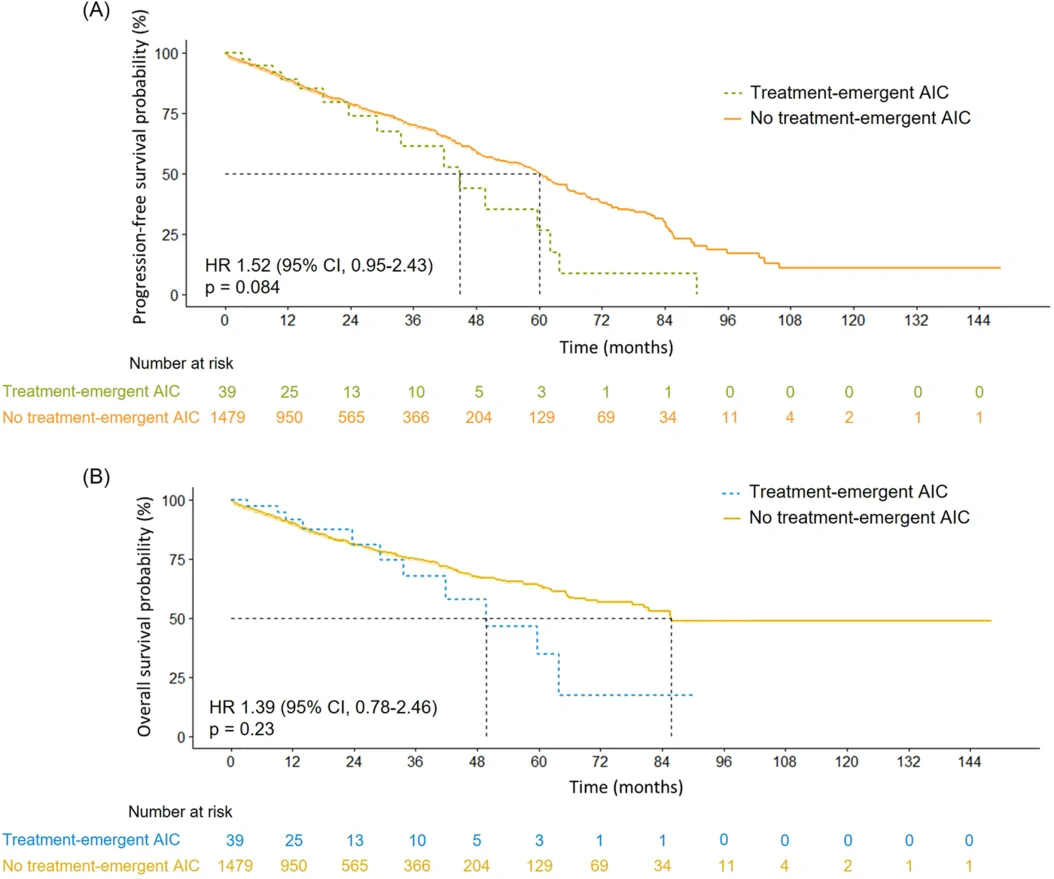
**Progression‐free survival (PFS) and overall survival (OS) in patients with and without treatment‐emergent autoimmune cytopenias (AIC).** Median PFS was 44.8 and 59.9 months for patients with and without treatment‐emergent AIC, respectively **(A)**. Median OS was 49.7 and 85.6 months for patients with and without treatment‐emergent AIC, respectively **(B)**. HR, hazard ratio.

In terms of AIC management, 97.3% of patients who developed treatment‐emergent AIC received an AIC‐directed treatment. According to the treating physician's choice, 61.1% of patients were treated with corticosteroids alone, while 19.5% received a combination of corticosteroids and rituximab. Few patients were treated with IVIG, steroids + IVIG, steroids + rituximab + IVIG, or IVIG + a thrombopoietin receptor agonist.

Among the 39 patients who developed treatment‐emergent AIC, 31 episodes were not considered to be associated with CLL progression, whereas 5 occurred in temporal association with disease progression. Two patients deceased from fulminant AIHA; however, the available data did not allow us to determine whether additional criteria of concomitant CLL progression were present. One additional patient permanently discontinued venetoclax because of AIC, although the presence of concomitant disease progression could not be reliably assessed.

Information on venetoclax management was available for most, but not all, patients. Dose modification data were available for 36 patients: venetoclax dose was reduced in 7 patients (6 permanently and 1 temporarily), whereas no dose reduction was required in the remaining 29 patients. Information on treatment interruption was available for 35 patients: venetoclax was temporarily interrupted in 13 patients and permanently discontinued in 6, whereas treatment was continued without interruption in 16 patients. Overall, among patients with available data, venetoclax was either continued without interruption or successfully resumed after a temporary interruption in 29 of 35 cases (83%).

Following AIC management, the episodes were considered resolved in 47.4% of patients, controlled in 34.2%, and active in 18.4%, with no significant differences between patients receiving venetoclax monotherapy and those receiving venetoclax‐based combinations. Specifically, in the monotherapy group, AIC were resolved, controlled, or active in 40%, 33.3%, or 26.7%, respectively, whereas in the combination group the rates were 52.2%, 34.8%, or 13%, respectively.

## DISCUSSION

In this study, through a global collaboration within the ERIC, we provide the largest analysis to date regarding the impact of treatment with venetoclax—given as single agent or as part of a combination regimen—on preexisting and treatment‐emergent AIC from a large cohort of patients with CLL, including both TN and R/R cases. We show that a history of preexisting AIC does not affect the outcome of patients with CLL treated with venetoclax‐based therapies, and that venetoclax‐containing regimens have an overall beneficial impact on the outcome of preexisting autoimmune phenomena. In addition, we observed that the administration of a venetoclax‐based combination regimen containing an anti‐CD20 mAb (either rituximab or obinutuzumab) and/or a BTKi exerts a more protective effect than venetoclax monotherapy toward the occurrence of new‐onset treatment‐emergent AIC. Notably, our analysis shows that AIC complications occurring during venetoclax‐based treatments can be successfully managed in most patients.

The clinical and biological characteristics of patients at treatment initiation, the treatment distribution and the overall therapeutic efficacy aligned with expectations for this patient cohort. Specifically, in line with treatment guidelines,^26^ most previously untreated patients received venetoclax in combination with an anti‐CD20 mAb—primarily obinutuzumab—whereas venetoclax monotherapy and the venetoclax plus rituximab combo were predominant in the R/R setting. In terms of treatment outcome, both PFS and OS were significantly longer in patients receiving venetoclax‐based treatment as a frontline therapy, not dissimilar to those reported in clinical trials.^27^, ^28^

The 11.7% rate of preexisting AIC observed in the present study is comparable with previously published results.^5^, ^6^, ^7^, ^8^, ^9^ In the present cohort, the preexisting AIC rate was significantly higher in patients with R/R disease compared to TN patients, consistent with previous reports indicating an increased incidence of autoimmune complications in heavily pretreated populations.^8^ In the current analysis, the presence of preexisting AIC did not associate with unfavorable prognostic parameters, including chromosomal aberrations and IGHV gene mutational status. Although this is in contrast with some previous findings,^8^, ^29^, ^30^ the discrepancy could be explained by the heterogeneity of patients across different cohorts and the overall low number of AIC events observed. Similarly, in the current analysis and consistent with our previous publication,^9^ a preexisting AIC did not have a significant impact on disease outcome in terms of PFS or OS.

Notably, during treatment with venetoclax, we observed only a few AIC recurrences (patients with resolved or controlled AIC at treatment initiation who were categorized as worsened during treatment). Moreover, we observed an overall AIC recovery in most patients who had a controlled or active autoimmune phenomena at treatment initiation. This observation is in line with our previously reported experience^9^ and was noted in patients treated with venetoclax both as a single agent and in combination, without meaningful differences between the groups. Given the high efficacy of venetoclax‐based treatments in both TN and R/R cohorts—as demonstrated by the high ORR and CR/CRi rates—we can infer that the overall beneficial effect on the management of AIC is most likely associated with effective control of the underlying CLL.

In the present study, the overall incidence of treatment‐emergent AIC was low (2.6%) among all venetoclax‐treated patients. It is important to highlight that—based on the study design and to allow for comparisons with our previous report—we considered only newly occurring treatment‐emergent AIC, excluding from this calculation all patients who have had a history of preexisting AIC at the time of treatment initiation. This type of analysis may underestimate the incidence of AIC episodes occurring during treatment compared to other studies in which all AIC—including possible relapses or exacerbations of preexisting AIC—were captured.

In line with our previous study^9^—where all venetoclax‐treated patients developing a treatment‐emergent AIC were carrying a *TP53* aberration—in the current analysis we also observed a higher proportion of *TP53* aberrations in patients who developed treatment‐emergent AIC, supporting the hypothesis that an adverse biology may favor the onset of newly occurring autoimmune complications.

Notably, the rate of treatment‐emergent AIC in our study was higher among patients treated with venetoclax monotherapy than among those receiving venetoclax‐based combination regimens. However, after adjustment for the main available prognostic variables, this association was attenuated and no longer reached statistical significance, suggesting that differences in baseline patient characteristics may have contributed to the observed association. This finding may be partially explained by treatment allocation, as single‐agent venetoclax was primarily administered to patients with R/R disease, whereas nearly all patients with TN CLL received venetoclax in combination with an anti‐CD20 mAb and/or a BTKi. Despite this, the lower unadjusted frequency of treatment‐emergent AIC observed among patients receiving anti‐CD20‐containing regimens and/or BTKi combinations remains biologically plausible. Indeed, the addition of an anti‐CD20 mAb may provide an immunosuppressive protective effect, while BTKi therapy has been shown to reduce the production of pathogenic autoantibodies and to decrease macrophage‐mediated clearance of autoantibody‐coated cells.^31^

Interestingly, patients who developed treatment‐emergent AIC achieved a significantly lower CR/CRi rate. However, the greater burden of *TP53* aberrations in this subgroup may have partly accounted for the lower response rates observed, suggesting that underlying disease biology may have contributed to this finding. Despite the lower CR/CRi rate, no differences in PFS and OS were observed between patients who did and did not develop treatment‐emergent AIC. Moreover, the overall outcome of treatment‐emergent AIC was favorable, with over 80% of patients achieving resolution or adequate control of the event through autoimmunity‐directed treatment, irrespective of the type of venetoclax‐based therapy received.

We acknowledge certain limitations to our study, in part imputable to its retrospective nature. Primarily, missing information, sampling, and attrition bias‐inherent problems could apply. Several baseline variables were not available for all patients because testing was not uniformly performed across participating centers. Consequently, analyses involving these variables should be interpreted with caution, as missing data may have reduced statistical power and introduced selection bias. We somewhat mitigated the aforementioned limitations by (i) collecting data on all consecutive patients with CLL undergoing venetoclax‐containing treatments in each participating center and (ii) using curation mechanisms that minimized missing information and ensured data quality. Also, collecting PFS in a real‐world study might be less robust than in controlled prospective trials: this was not the primary objective of the study, and we nevertheless did provide the data, which should be interpreted with caution. Furthermore, although survival outcomes were estimated using standard Kaplan–Meier methodology, the median follow‐up duration was relatively short in relation to the long‐term survival estimates reported. As a result, estimates obtained at later time points are based on a limited number of patients at risk and should be interpreted accordingly.

Altogether, our data indicate that venetoclax‐based treatments have a favorable impact on the course and outcome of CLL‐associated preexisting AIC. Treatment‐emergent AIC are uncommon and appear to occur more frequently with venetoclax monotherapy than with combination regimens. Importantly, these events are generally manageable and do not seem to compromise treatment efficacy. Given their overall rarity—particularly in patients receiving time‐limited venetoclax combinations—and the high likelihood of successful management, these complications should not influence treatment decision‐making for either TN or R/R patients with CLL.

## AUTHOR CONTRIBUTIONS

**Candida Vitale**: Writing—original draft; writing—review and editing; conceptualization; methodology; investigation; validation; formal analysis. **Thomas Chatzikonstantinou**: Methodology; data curation; formal analysis; project administration; writing—review and editing; investigation. **Christina Papangelou**: Data curation; formal analysis; writing—review and editing; investigation. **Francesca Perutelli**: Investigation; writing—review and editing. **Maria Chiara Montalbano**: Investigation; writing—review and editing. **Giulia Bondielli**: Investigation; writing—review and editing. **Elia Boccellato**: Investigation; writing—review and editing. **Salem Alshemmari**: Investigation; writing—review and editing. **Francesco Angotzi**: Investigation; writing—review and editing. **Miguel Arguello‐Tomas**: Investigation; writing—review and editing. **Tomas Arpas**: Investigation; writing—review and editing. **Sandra Bašić‐Kinda**: Investigation; writing—review and editing. **Sotiria Besikli‐Dimou**: Investigation; writing—review and editing. **Łukasz Bołkun**: Investigation; writing—review and editing. **Tomasz Buczek**: Investigation; writing—review and editing. **Aleksandra Butrym**: Investigation; writing—review and editing. **Katherine Chapis**: Investigation; writing—review and editing. **Sofia Chatzileontiadou**: Investigation; writing—review and editing. **Rosa Collado**: Investigation; writing—review and editing. **Raul Cordoba Mascuñano**: Investigation; writing—review and editing. **Lorenzo De Paoli**: Investigation; writing—review and editing. **Marina Deodato**: Investigation; writing—review and editing. **Maria Dimou**: Investigation; writing—review and editing. **David Donaldson**: Investigation; writing—review and editing. **Joanna Drozd‐Sokołowska**: Investigation; writing—review and editing. **Salem Elias**: Investigation; writing—review and editing. **Andrea Ferrario**: Investigation; writing—review and editing. **Alberto Fresa**: Investigation; writing—review and editing. **Anna Maria Frustaci**: Investigation; writing—review and editing. **Rocío García‐Serra**: Investigation; writing—review and editing. **Ajay Gogia**: Investigation; writing—review and editing. **Isabel González‐Gascón‐y‐Marín**: Investigation; writing—review and editing. **Vasko Graklanov**: Investigation; writing—review and editing. **Maria Hryniewiecka**: Investigation; writing—review and editing. **Zhu Huayuan**: Investigation; writing—review and editing. **Elzbieta Iskierka‐Jażdżewska**: Investigation; writing—review and editing. **Paula Jablonowska‐Babij**: Investigation; writing—review and editing. **Ozren Jaksic**: Investigation; writing—review and editing. **Elzbieta Kalicińska**: Investigation; writing—review and editing. **Maria Kislova**: Investigation; writing—review and editing. **Maya Koren‐Michowitz**: Investigation; writing—review and editing. **Epameinondas Koumpis**: Investigation; writing—review and editing. **Zuzana Kubová**: Investigation; writing—review and editing. **Jorge Labrador**: Investigation; writing—review and editing. **Pietro Lauzzana**: Investigation; writing—review and editing. **Shai Levi**: Investigation; writing—review and editing. **Francesca Martini**: Investigation; writing—review and editing. **Mariia Mikhaleva**: Investigation; writing—review and editing. **Eva Minga**: Investigation; writing—review and editing. **Riccardo Moia**: Investigation; writing—review and editing. **Elżbieta Morawiec‐Szymonik**: Investigation; writing—review and editing. **Marta Morawska**: Investigation; writing—review and editing. **Femke V. M. Mulder**: Investigation; writing—review and editing. **Roberta Murru**: Investigation; writing—review and editing. **Jacopo Olivieri**: Investigation; writing—review and editing. **Ewa Paszkiewicz‐Kozik**: Investigation; writing—review and editing. **Lata Rani**: Investigation; writing—review and editing. **Josep Maria Roncero**: Investigation; writing—review and editing. **Wojciech Salamon**: Investigation; writing—review and editing. **Alessandro Sanna**: Investigation; writing—review and editing. **Lydia Scarfò**: Investigation; writing—review and editing. **Tereza Shokralla**: Investigation; writing—review and editing. **Martin Šimkovič**: Investigation; writing—review and editing. **Matevž Škerget**: Investigation; writing—review and editing. **Emil Spasov**: Investigation; writing—review and editing. **Antigoni Tranidou**: Investigation; writing—review and editing. **Jagoda Tryc‐Szponder**: Investigation; writing—review and editing. **Marzia Varettoni**: Investigation; writing—review and editing. **Andrea Visentin**: Investigation; writing—review and editing. **George Vrachiolias**: Investigation; writing—review and editing. **David Waddell**: Investigation; writing—review and editing. **Ewa Wasik‐Szczepanek**: Investigation; writing—review and editing. **Kamil Wdowiak**: Investigation; writing—review and editing. **Mateusz Wilk**: Investigation; writing—review and editing. **Kamil Wiśniewski**: Investigation; writing—review and editing. **Magdalena Witkowska**: Investigation; writing—review and editing. **Mohamed Yassin**: Investigation; writing—review and editing. **Seyma Yıldız**: Investigation; writing—review and editing. **Klaudia Zielonka**: Investigation; writing—review and editing. **Nil Albiol**: Investigation; writing—review and editing. **Jennifer R. Brown**: Investigation; writing—review and editing. **Benedetto Bruno**: Investigation; writing—review and editing. **Mark Catherwood**: Investigation; writing—review and editing. **Anastasia Chatzidimitriou**: Investigation; writing—review and editing. **Michael Doubek**: Investigation; writing—review and editing. **Gianluca Gaidano**: Investigation; writing—review and editing. **Alessandro Gozzetti**: Investigation; writing—review and editing. **Ritu Gupta**: Investigation; writing—review and editing. **Eleftheria Hatzimichael**: Investigation; writing—review and editing. **Yair Herishanu**: Investigation; writing—review and editing. **Krzysztof Jamroziak**: Investigation; writing—review and editing. **Ioannis Kotsianidis**: Investigation; writing—review and editing. **Luca Laurenti**: Investigation; writing—review and editing. **Eugene Nikitin**: Investigation; writing—review and editing. **Maria Papaioannou**: Investigation; writing—review and editing. **Tomáš Papajik**: Investigation; writing—review and editing. **Martin Špaček**: Investigation; writing—review and editing. **Niki Stavroyianni**: Investigation; writing—review and editing. **Josephine M. I. Vos**: Investigation; writing—review and editing. **Tomasz Wróbel**: Investigation; writing—review and editing. **Münci Yağcı**: Investigation; writing—review and editing. **Arnon P. Kater**: Investigation; writing—review and editing. **Matthew Davids**: Investigation; writing—review and editing. **Petra Langerbeins**: Investigation; writing—review and editing. **Kostas Stamatopoulos**: Writing—review and editing; supervision; investigation; methodology. **Paolo Ghia**: Supervision; writing—review and editing; investigation; methodology. **Marta Coscia**: Conceptualization; methodology; investigation; validation; formal analysis; writing—review and editing; writing—original draft; supervision.

## CONFLICT OF INTEREST STATEMENT

Marta Coscia—research support: AbbVie, GSK, and Johnson & Johnson; advisory board/speaker's bureau: AbbVie, AstraZeneca, Behring, BeOne, Bristol Myers Squibb, GSK, Johnson & Johnson, and Lilly; travel support: Johnson & Johnson, BeOne, AstraZeneca, and AbbVie. Matthew Davids—consulting: AbbVie, Ascentage Pharma, AstraZeneca, BeiGene/BeOne, Bristol‐Myers Squibb, Eli Lilly, Galapagos, Genentech, Genmab, Janssen, Merck, MEI Pharma, Nuvalent, and Schrödinger; institutional research support: Ascentage, AstraZeneca, and MEI Pharma; royalties: UpToDate. Gianluca Gaidano—research support: Gilead; advisory board/speaker's bureau: AbbVie, AstraZeneca, BeOne, Hikma, Incyte, Johnson & Johnson, and Lilly. Eleftheria Hatzimichael—honoraria: Roche, AstraZeneca, Johnson & Johnson, Genesis, and AbbVie; advisory board/consultancy: Roche, Johnson & Johnson, Genesis, AbbVie, MSD, and AstraZeneca. Petra Langerbeins—research grants: Janssen and BeOne; consultancy fees/advisory board: Janssen, BeOne, AstraZeneca, and AbbVie; travel support: Janssen, BeOne, AstraZeneca, and AbbVie. Riccardo Moia—advisory board and speaker's honoraria: Johnson & Johnson, AbbVie, AstraZeneca, and BeOne. Martin Šimkovič—honoraria, advisory board membership, travel grants: AbbVie, AstraZeneca, BeOne, Eli Lilly, Johnson & Johnson, MSD, and Swixx Biopharma. Martin Špaček—advisory board/consultancy: AbbVie, Eli Lilly, Janssen, and Swixx; honoraria: AbbVie, AstraZeneca, Eli Lilly, Janssen, and Swixx; travel support: AbbVie, AstraZeneca, Eli Lilly, Janssen, and Swixx. Marzia Varettoni—honoraria and travel expense: AbbVie, AstraZeneca, BeOne, and Johnson & Johnson; speaker bureau and travel expenses: Lilly. Candida Vitale—consultancy fees/advisory board: Johnson & Johnson, AbbVie, and BeOne; travel support: Johnson & Johnson, AbbVie, BeOne, AstraZeneca, and Lilly; speakers' bureau: Lilly. Josephine M.I. Vos—research support: BeiGene, AbbVie/Genmab; advisory board/consultancy fees: Sanofi and Janssen; speaker fees: BMS, Sanofi, BeiGene, Novartis, and Amgen. Kamil Wiśniewski—honoraria: AbbVie, BeiGene, AstraZeneca, Amgen, Gilead, and Johnson & Johnson; travel support: AbbVie, BeiGene, Amgen, and Roche. The remaining authors have no conflicts of interest to declare.

## ETHICS STATEMENT

The study was conducted in accordance with the Declaration of Helsinki. The study was reviewed and approved by the Institutional Review Board (IRB) “Comitato Etico Interaziendale A.O.U. Città della Salute e della Scienza di Torino – A.O. Ordine Mauriziano – A.S.L. Città di Torino” (reference number: 0051208 ‐ 21/Apr/2023). Furthermore, local approval or formal authorization was obtained at all other participating institutions in accordance with their respective local ethical standards and regulatory requirements.

## FUNDING

Open access publishing facilitated by Universita degli Studi dell'Insubria, as part of the Wiley ‐ CRUI‐CARE agreement.

## Supporting information

Supporting Information.

## Data Availability

De‐identified individual participant data underlying the results reported in this article will be made available upon reasonable request following publication, subject to approval by the investigators and in compliance with applicable ethical approvals and data protection regulations. Requests for access to the data, including original data, should be addressed to the corresponding author.
