# Supplementary material for: Impact of venetoclax‐based therapies on CLL‐associated autoimmune cytopenias: A retrospective study conducted by ERIC
Source: Hemasphere. 2026 Nov 6;10(11):e70522. doi: 10.1002/hem3.70522 (PMC13743197; doi:10.1002/hem3.70522)
Supplement: Supplementary file 1 [file HEM3-10-e70522-s001.docx]

**Supplemental material**

**Impact of venetoclax-based therapies on CLL-associated autoimmune cytopenias: a retrospective study conducted by ERIC**

**Supplemental Table S1. AIC definitions shared with participating centers^1,2^.**

**Autoimmune hemolytic anemia (AIHA)**

Presence of all the following:

- Hb levels ≤11 g/dL, in the absence of any cytotoxic treatment in the preceding month or other etiology identified;
- evidence of an underlying autoimmune mechanism, such as a positive direct antiglobulin test (DAT) or the presence of cold agglutinin, after exclusion of alternatives (i.e., delayed hemolytic transfusion reaction);
- presence of one or more laboratory marker of hemolysis (high reticulocyte count, low serum haptoglobin levels, increased serum lactate dehydrogenase, or bilirubin levels).

**Immune Thrombocytopenia (ITP)**

Presence of all the following:

- otherwise unexplained and sudden fall in platelet count (<100 × 10^9^/L), in the presence of normal bone marrow function (normal or increased number of megakaryocytes at bone marrow examination);
- no evidence of splenomegaly and no cytotoxic treatments within the last month;
- exclusion of other possible causes of thrombocytopenia (e.g., drug induced thrombocytopenia, infections, thrombotic thrombocytopenic purpura, disseminated intravascular coagulation).

In patients who were not studied with a bone marrow biopsy, a rapid response to steroid treatment was used as an *ex juvantibus* criterion to support the hypothesis of ITP.

**Pure red blood cell aplasia (PRCA)**

Presence of all the following:

- Hb levels ≤11 g/dL, in the absence of hemolysis;
- absolute reticulocytopenia, in the absence of thrombocytopenia or neutropenia;
- exclusion of other causes of red cell aplasia (i.e., viral infections or thymoma).

**Supplemental Table S2. Categorization of the status and outcome of preexisting and treatment-emergent AIC^2^.**

| **Pre- existing AIC status at the start of targeted drug** | |
| --- | --- |
| *Resolved* | Complete blood count normalization, no signs of hemolysis and no transfusion support or other AIC-directed treatment (including steroids or TPO mimetics) |
| *Controlled* | Blood counts stable over time but not reaching the normal values, or presence of subclinical hemolysis (hemoglobin within normal limits but high reticulocyte count and/or low serum haptoglobin levels) |
| *Active* | Blood counts decreasing, requiring treatment change or additional medical treatment |
| **Pre-existing AIC outcome during targeted drug treatment** | |
| *Resolved* | Complete blood count normalization, no signs of hemolysis and no transfusion support or other AIC-directed treatment (including steroids or TPO mimetics) |
| *Improved* | Count recovery without complete normalization, or presence of persistent signs of subclinical hemolysis (hemoglobin within normal limits but high reticulocyte count and/or low serum haptoglobin levels) |
| *Stable* | Absence of relevant changes in blood parameters |
| *Worsened* | Worsening of cytopenia, or need of additional therapy to control AIC |
| **Treatment-emergent AIC outcome during targeted drug treatment** | |
| *Resolved* | Complete blood count normalization, no signs of hemolysis and no transfusion support or other AIC-directed treatment (including steroids or TPO mimetics) |
| *Controlled* | Blood counts stable over time but not reaching the normal values, or presence of subclinical hemolysis (hemoglobin within normal limits but high reticulocyte count and/or low serum haptoglobin levels) |
| *Active* | Blood counts decreasing, requiring treatment change or additional medical treatment |

**Supplemental Table S3. Characteristics of patients with and without preexisting AIC at the start of venetoclax-based treatment.**

|  | **Preexisting AIC** | | **No preexisting AIC** | | **P value** |
| --- | --- | --- | --- | --- | --- |
|  | No. evaluable patients |  | No. evaluable patients |  |  |
| Male sex, number (%) | 202 | 148 (73.3%) | 1518 | 1008 (66.4%) | NS |
| Age, years, median (range) | 202 | 69 (33-86) | 1518 | 68 (27-92) | NS |
| Binet stage, number (%)  A  B  C | 164 | 23 (14%)  37 (22.6%)  104 (63.4%) | 1069 | 149 (13.9%)  473 (44.2%)  447 (41.8%) | NS |
| CIRS score, median (range) | 129 | 7 (1-17) | 906 | 5 (0-30) | NS |
| β_2_-microglobulin, mg/L, median (range) | 102 | 4.1 (1.1-10) | 626 | 3.9 (0.9-17.9) | NS |
| Unmutated IGHV status, number (%) | 126 | 87 (69%) | 879 | 631 (71.8%) | NS |
| del(13q), number (%) | 124 | 74 (59.7%) | 873 | 419 (48%) | NS |
| del(11q), number (%) | 146 | 40 (27.4%) | 988 | 262 (26.5%) | NS |
| tris(12), number (%) | 123 | 18 (14.6%) | 837 | 178 (21.3%) | NS |
| del(17p), number (%) | 163 | 31 (19%) | 1093 | 205 (18.8%) | NS |
| Mutated *TP53*, number (%) | 112 | 22 (19.6%) | 805 | 156 (19.4%) | NS |
| *TP53* aberration, number (%) | 118 | 43 (36.4%) | 803 | 281 (33.7%) | NS |
| Hypogammaglobulinemia (IgG <300 mg/dL), number (%) | 128 | 19 (14.8%) | 747 | 75 (10%) | NS |
| DAT positivity | 104 | **66 (63.5%)** | 618 | **64 (10.4%)** | **p<0.001** |
| DAT specificity, number (%)  anti-IgG  anti-C3  anti-IgG and anti-C3  other | 61 | 35 (57.4%)  2 (3.3%)  22 (36.1%)  2 (3.3%) | 37 | 124 (64.9%)  4 (10.8%)  9 (24.3%)  0 | NS |
| History of other type of autoimmune phenomena, number (%) | 153 | 15 (9.8%) | 866 | 59 (6.8%) | NS |
| Treatment-naïve patients, number (%) | 202 | **52 (25.7%)** | 1518 | **546 (36%)** | **p=0.005** |
| Number of previous therapies, median (range) | 202 | 1 (0-8) | 1518 | 1 (0-13) | NS |

Categorical variables were compared using the χ² test and continuous variables using Student’s t-test. Only statistically significant p-values are reported; all other comparisons were not statistically significant (NS).

Abbreviations: AIC, autoimmune cytopenia; CIRS, cumulative illness rating scale; IGHV, immunoglobulin heavy variable; DAT, direct antiglobulin test.

**Supplemental Table S4. Characteristics of treatment-naïve patients with and without preexisting AIC at the start of venetoclax-based treatment.**

|  | **Preexisting AIC** | | **No preexisting AIC** | | **P value** |
| --- | --- | --- | --- | --- | --- |
|  | No. evaluable patients |  | No. evaluable patients |  |  |
| Male sex, number (%) | 52 | 38 (73.1%) | 546 | 352 (64.5%) | NS |
| Age, years, median (range) | 52 | 69 (33-84) | 546 | 66 (30-90) | NS |
| Binet stage, number (%)  A  B  C | 46 | 11 (23.9%)  7 (15.2%)  28 (60.9%) | 430 | 71 (16.5%)  195 (45.3%)  164 (38.1%) | NS |
| CIRS score, median (range) | 56 | 6 (1–15) | 322 | 5 (0–28) | NS |
| β_2_-microglobulin, mg/L, median (range) | 39 | 3.8 (1.1-8.5) | 298 | 3.7 (0.9-14.0) | NS |
| Unmutated IGHV status, number (%) | 34 | 22 (64.7%) | 332 | 204 (61.4%) | NS |
| del(13q), number (%) | 39 | 23 (59%) | 383 | 193 (50.4%) | NS |
| del(11q), number (%) | 42 | 6 (14.3%) | 401 | 68 (17%) | NS |
| tris(12), number (%) | 39 | 8 (20.5%) | 363 | 86 (23.7%) | NS |
| del(17p), number (%) | 46 | 5 (10.9%) | 438 | 32 (7.3%) | NS |
| Mutated *TP53*, number (%) | 34 | 5 (14.7%) | 353 | 26 (7.4%) | NS |
| *TP53* aberration, number (%) | 38 | 8 (22.9%) | 330 | 43 (13%) | NS |
| Hypogammaglobulinemia (IgG <300 mg/dL), number (%) | 24 | 3 (12.5%) | 304 | 29 (9.5%) | NS |
| DAT positivity | **34** | **22 (64.7%)** | **275** | **35 (12.7%)** | **p < 0.001** |
| DAT specificity, number (%)  anti-IgG  anti-C3  anti-IgG and anti-C3  other | 22 | 13 (59.1%)  0  8 (36.4%)  1 (4.5%) | 16 | 12 (75%)  2 (12.5%)  2 (12.5%)  0 | NS |
| History of other type of autoimmune phenomena, number (%) | 37 | 3 (8.1%) | 385 | 26 (6.8%) | NS |

Categorical variables were compared using the χ² test and continuous variables using Student’s t-test. Only statistically significant p-values are reported; all other comparisons were not statistically significant (NS).

Abbreviations: AIC, autoimmune cytopenia; CIRS, cumulative illness rating scale; IGHV, immunoglobulin heavy variable; DAT, direct antiglobulin test.

**Supplemental Table S5. Characteristics of relapsed/refractory patients with and without preexisting AIC at the start of venetoclax-based treatment.**

|  | **Preexisting AIC** | | **No preexisting AIC** | | **P value** |
| --- | --- | --- | --- | --- | --- |
|  | No. evaluable patients |  | No. evaluable patients |  |  |
| Male sex, number (%) | 150 | 110 (73.3%) | 972 | 656 (67.5%) | NS |
| Age, years, median (range) | 150 | 70 (47-86) | 972 | 69 (27-92) | NS |
| Binet stage, number (%)  A  B  C | 117 | 13 (11.1%)  30 (25.6%)  74 (63.2%) | 639 | 79 (12.4%)  281 (44%)  279 (43.7%) | NS |
| CIRS score, median (range) | 73 | 8 (2–17) | 584 | 6 (0–30) | NS |
| β_2_-microglobulin, mg/L, median (range) | 63 | 4.3 (1.5–10.0) | 430 | 4.0 (1.1–17.9) | NS |
| Unmutated IGHV status, number (%) | 92 | 65 (70.7%) | 547 | 427 (78.1%) | NS |
| del(13q), number (%) | 85 | 51 (60%) | 490 | 225 (45.9%) | NS |
| del(11q), number (%) | 103 | 34 (33%) | 589 | 194 (32.9%) | NS |
| tris(12), number (%) | 84 | 10 (11.9%) | 475 | 91 (19.2%) | NS |
| del(17p), number (%) | 117 | 26 (22.2%) | 658 | 174 (26.4%) | NS |
| Mutated *TP53*, number (%) | 77 | 17 (22.1%) | 454 | 134 (29.5%) | NS |
| *TP53* aberration, number (%) | 82 | 35 (42.7%) | 477 | 231 (48.4%) | NS |
| Hypogammaglobulinemia (IgG <300 mg/dL), number (%) | 104 | 16 (15.4%) | 443 | 46 (10.4%) | NS |
| DAT positivity | **68** | **42 (61.8%)** | **343** | **28 (8.2%)** | **p < 0.001** |
| DAT specificity, number (%)  anti-IgG  anti-C3  anti-IgG and anti-C3  other | 38 | 22 (57.9%)  2 (5.3%)  13 (34.2%)  1 (2.6%) | 20 | 13 (65%)  1 (5%)  6 (30%)  0 | NS |
| History of other type of autoimmune phenomena, number (%) | 116 | 12 (10.3%) | 481 | 33 (6.9%) | NS |
| Number of previous therapies, median (range) | 150 | 2 (1-8) | 972 | 2 (1-13) | NS |

Categorical variables were compared using the χ² test and continuous variables using Student’s t-test. Only statistically significant p-values are reported; all other comparisons were not statistically significant (NS).

Abbreviations: AIC, autoimmune cytopenia; CIRS, cumulative illness rating scale; IGHV, immunoglobulin heavy variable; DAT, direct antiglobulin test.

**Supplemental Table S6. Univariable and multivariable Cox proportional hazards regression analysis of prognostic factors for overall survival in treatment-naïve patients.**

| **Characteristic** | **Univariable anlysis** | | | | **Multivariable analysis** | | |
| --- | --- | --- | --- | --- | --- | --- | --- |
|  | N | HR | 95% CI | P value | HR | 95% CI | P value |
| Age at CLL diagnosis | 565 | 1.07 | 1.03, 1.11 | <0.001 | 1.09 | 1.02, 1.15 | 0.009 |
| IGHV gene status | 366 |  |  |  |  |  |  |
| Mutated |  | Ref. |  |  | Ref. |  |  |
| Unmutated |  | 0.43 | 0.15, 1.23 | 0.11 | 0.58 | 0.17, 1.96 | 0.4 |
| TP53 aberration status | 364 |  |  |  |  |  |  |
| Mutated |  | Ref. |  |  | Ref. |  |  |
| Unmutated |  | 0.32 | 0.11, 0.87 | 0.026 | 0.85 | 0.18, 4.08 | 0.8 |

Multivariable Cox regression analyses were performed separately in treatment-naïve and relapsed/refractory patients. Covariates included age, IGHV mutational status, TP53 aberration status and previous treatment burden (where applicable). DAT positivity and hypogammaglobulinemia were excluded because of missing data.

Abbreviations: CI, Confidence Interval; HR, Hazard Ratio; Ref, reference category.

**Supplemental Table S7. Univariable and multivariable Cox proportional hazards regression analysis of prognostic factors for overall survival in relapsed/refractory patients.**

| **Characteristic** | **Univariable anlysis** | | | | **Multivariable analysis** | | |
| --- | --- | --- | --- | --- | --- | --- | --- |
|  | N | HR | 95% CI | P value | HR | 95% CI | P value |
| Age at CLL diagnosis | 1077 | 1.02 | 1.01, 1.03 | <0.001 | 1.03 | 1.01, 1.05 | 0.011 |
| Lines before Venetoclax | 1120 | 1.25 | 1.18, 1.32 | <0.001 | 1.20 | 1.10, 1.31 | <0.001 |
| Treatment status | 1073 |  |  |  |  |  |  |
| Treated |  | Ref. |  |  | Ref. |  |  |
| Untreated |  | 2.06 | 1.06, 4.01 | 0.032 | 0.96 | 0.30, 3.04 | >0.9 |
| IGHV gene status | 639 |  |  |  |  |  |  |
| Mutated |  | Ref. |  |  | Ref. |  |  |
| Unmutated |  | 1.90 | 1.29, 2.81 | 0.001 | 1.26 | 0.79, 2.03 | 0.3 |
| TP53 aberration status | 559 |  |  |  |  |  |  |
| Mutated |  | Ref. |  |  | Ref. |  |  |
| Unmutated |  | 0.61 | 0.45, 0.83 | 0.001 | 0.82 | 0.57, 1.20 | 0.3 |

Multivariable Cox regression analyses were performed separately in treatment-naïve and relapsed/refractory patients. Covariates included age, IGHV mutational status, TP53 aberration status and previous treatment burden (where applicable). DAT positivity and hypogammaglobulinemia were excluded because of missing data.

Abbreviations: CI, Confidence Interval; HR, Hazard Ratio; Ref, reference category.

**Supplemental Table S8. Univariable and multivariable logistic regression analysis of factors associated with treatment-emergent AIC.**

| **Characteristic** | **Univariable anlysis** | | | | **Multivariable analysis** | | |
| --- | --- | --- | --- | --- | --- | --- | --- |
|  | N | OR | 95% CI | P value | OR | 95% CI | P value |
| Age at CLL diagnosis | 1452 | 1.00 | 0.97, 1.03 | >0.9 |  |  |  |
| Lines before Venetoclax | 1518 | 1.28 | 1.09, 1.47 | 0.001 | 1.21 | 0.99, 1.45 | 0.042 |
| Treatment status | 1439 |  |  |  |  |  |  |
| Treated |  | Ref. |  |  |  |  |  |
| Untreated |  | 2.68 | 0.15, 13.9 | 0.3 |  |  |  |
| IGHV gene status | 880 |  |  |  |  |  |  |
| Mutated |  | Ref. |  |  |  |  |  |
| Unmutated |  | 1.42 | 0.56, 4.35 | 0.5 |  |  |  |
| TP53 aberration status | 808 |  |  |  |  |  |  |
| Mutated |  | Ref. |  |  | Ref. |  |  |
| Unmutated |  | 0.43 | 0.19, 0.94 | 0.034 | 0.69 | 0.29, 1.61 | 0.4 |
| Type of treatment | 1518 |  |  |  |  |  |  |
| Venetoclax - combination |  | Ref. |  |  | Ref. |  |  |
| Venetoclax - single |  | 2.26 | 1.14, 4.35 | 0.016 | 2.23 | 0.89, 5.57 | 0.086 |

Abbreviations: CI, Confidence Interval; OR, Odds Ratio; Ref, reference category.

**Supplemental Table S9. Univariable and multivariable Cox proportional hazards regression analysis of the association between treatment-emergent AIC and overall survival.**

| **Characteristic** | **Univariable anlysis** | | | | **Multivariable analysis** | | |
| --- | --- | --- | --- | --- | --- | --- | --- |
|  | N | HR | 95% CI | P value | HR | 95% CI | P value |
| Age at CLL diagnosis | 1451 | 1.02 | 1.01, 1.03 | <0.001 | 1.03 | 1.01, 1.05 | <0.001 |
| Lines before Venetoclax | 1516 | 1.32 | 1.26, 1.39 | <0.001 | 1.29 | 1.18, 1.41 | <0.001 |
| Treatment status | 1437 |  |  |  |  |  |  |
| Treated |  | Ref. |  |  | Ref. |  |  |
| Untreated |  | 2.33 | 1.15, 4.71 | 0.018 | 1.47 | 0.53, 4.04 | 0.5 |
| IGHV gene status | 880 |  |  |  |  |  |  |
| Mutated |  | Ref. |  |  | Ref. |  |  |
| Unmutated |  | 1.74 | 1.20, 2.54 | 0.004 | 1.13 | 0.70, 1.83 | 0.6 |
| TP53 aberration status | 807 |  |  |  |  |  |  |
| Mutated |  | Ref. |  |  | Ref. |  |  |
| Unmutated |  | 0.40 | 0.29, 0.55 | <0.001 | 0.75 | 0.50, 1.13 | 0.2 |
| Treatment-emergent AIC during Venetoclax | 1516 |  |  |  |  |  |  |
| No |  | Ref. |  |  |  |  |  |
| Yes |  | 1.36 | 0.74, 2.48 | 0.3 |  |  |  |

Abbreviations: CI, Confidence Interval; HR, Hazard Ratio; Ref, reference category.

**References**

1. Vitale C, Montalbano MC, Salvetti C, et al. Autoimmune Complications in Chronic Lymphocytic Leukemia in the Era of Targeted Drugs. Cancers (Basel). 2020;12(2).
2. Vitale C, Salvetti C, Griggio V, et al. Preexisting and treatment-emergent autoimmune cytopenias in patients with CLL treated with targeted drugs. Blood. Jun 24 2021;137(25):3507-3517.
